# Network Mechanisms Underlying the Regional Diversity of Variance and Time Scales of the Brain's Spontaneous Activity Fluctuations

**DOI:** 10.1523/JNEUROSCI.1699-24.2024

**Published:** 2025-01-22

**Authors:** Adrián Ponce-Alvarez

**Affiliations:** ^1^Department of Mathematics, Polytechnic University of Catalonia, Barcelona 08028, Spain; ^2^Institut de Matemàtiques de la UPC - Barcelona Tech (IMTech), Barcelona 08028, Spain; ^3^Centre de Recerca Matemàtica, Barcelona 08193, Spain

**Keywords:** brain activity scales, hierarchies, human connectome, human fMRI, structure‒function relationships, whole-brain models

## Abstract

The brain's activity fluctuations have different temporal scales across the brain regions, with associative regions displaying slower timescales than sensory areas. This hierarchy of timescales has been shown to correlate with both structural brain connectivity and intrinsic regional properties. Here, using publicly available human resting-state fMRI and dMRI data, it was found that, while more structurally connected brain regions presented activity fluctuations with longer timescales, their activity fluctuations presented lower variance. The opposite relationships between the structural connectivity and the variance and temporal scales of resting-state fluctuations, respectively, were not trivially explained by simple network propagation principles. To understand these structure–function relationships, two commonly used whole-brain models were studied, namely, the Hopf and Wilson–Cowan models. These models use the brain's connectome to couple local nodes (representing brain regions) displaying noise-driven oscillations. The models show that the variance and temporal scales of activity fluctuations can oppositely relate to connectivity within specific parameter regions, even when all nodes have the same intrinsic dynamics—but also when intrinsic dynamics are constrained by the myelinization-related macroscopic gradient. These results show that, setting aside intrinsic regional differences, connectivity and network state are sufficient to explain the regional differences in fluctuations’ scales. State dependence supports the vision that structure–function relationships can serve as biomarkers of altered brain states. Finally, the results indicate that the hierarchies of timescales and variances reflect a balance between stability and responsivity, with greater and faster responsiveness at the network periphery, while the network core ensures overall system robustness.

## Significance Statement

Brain regions exhibit activity fluctuations at different temporal scales, with associative areas displaying slower timescales than sensory areas. This hierarchical organization is shaped by both large-scale connectivity and local properties. This study demonstrates that the variance of fluctuations is also hierarchically organized but, in contrast to timescales, it decreases as a function of structural connectivity. Whole-brain models show that the hierarchies of timescales and variances jointly emerge within specific parameter regions, indicating a state dependence that could serve as a biomarker for brain states and disorders. Furthermore, these hierarchies link to the responsivity of different network parts, with greater and faster responsiveness at the network periphery and more stable dynamics at the core, achieving a balance between stability and responsiveness.

## Introduction

The brain's activity fluctuations have different temporal scales across the brain regions, with higher-order associative regions displaying slower fluctuations than lower-order sensory areas (Kiebel et al., 2008; Honey et al., 2012; Murray et al., 2014; Gollo et al., 2017; Fallon et al., 2020; Ito et al., 2020; Raut et al., 2020). The regional variation of timescales has been measured with diverse recording techniques, such as electrophysiology (Honey et al., 2012; Murray et al., 2014), fMRI (Fallon et al., 2020; Manea et al., 2022), and MEG (Demirtaş et al., 2019). Notably, the organization of timescales is altered in the case of brain disorders (Watanabe et al., 2019), indicating a potential use as biomarker of neuropsychiatric disorders. Previous work has shown that both connectivity and local properties shape this so-called hierarchy of timescales. On the one hand, the number of spines on pyramidal neurons correlates with the hierarchy of timescales (Elston, 2003, 2007; Chaudhuri et al., 2015), so as the gradients of gene expression involving synapses and cell types (Burt et al., 2018). On the other hand, the timescale of human and mouse resting-state (rs) fMRI dynamics increases with structural connectivity strength, i.e., more strongly connected regions exhibit slower dynamics (Sethi et al., 2017; Fallon et al., 2020). The functional implications of a hierarchy of neural timescales across the brain are tied to the functional specialization of brain regions, with sensory neural circuits operating on short timescales to rapidly encode external stimuli, while higher-association circuits work over longer timescales, allowing for the accumulation and integration of information from diverse sources (Honey et al., 2012).

Though most fMRI studies use *z*-score normalized time series, previous studies have revealed interesting properties of the signal variability. Indeed, differences in the variances of activity fluctuations have been used to characterize aging (Garrett et al., 2011), brain states (Fulcher et al., 2013), neuropsychiatric disorders (Henderson et al., 2023), task activity (He, 2013; Ponce-Alvarez et al., 2015), and time-varying functional connectivity (Glomb et al., 2018). Notably, the standard deviation of fMRI signals has been proposed as a biomarker to classify schizophrenia (Kaufmann et al., 2015; Xie et al., 2018; Bryant et al., 2024) and autism (Easson and McIntosh, 2019; Deco et al., 2013; Bryant et al., 2024). From a theoretical perspective, there exist a deep link between a system's equilibrium fluctuations and its response to external forces, i.e., the fluctuation–dissipation theorem (Gardiner, 2004). In other words, if the susceptibility of a system to external change is large, then the fluctuations about equilibrium are expected to be large. It is, however, unknown whether the variance of the brain's spontaneous activity is hierarchically organized and how it relates to structural connectivity.

The present study analyzes the relationship between timescales, variances (i.e., magnitude scales), and structural connectivity using publicly available human rs-fMRI and dMRI data, while also exploring the underlying mechanisms through connectome-based whole-brain models. The results suggest that hierarchies in the variance and temporal scales of spontaneous activity fluctuations emerge in a nontrivial way from the interplay between connectivity and the system's dynamical state.

## Materials and Methods

### fMRI and diffusion-weighted imaging data

This study uses publicly available rs-fMRI data from the Human Connectome Project (HCP; Van Essen et al., 2013). The participants were scanned on a 3 T connectome-Skyra scanner (Siemens). The rs-fMRI data was acquired for ∼15 min, with eyes open and relaxed fixation on a dark background. The HCP website (https://www.humanconnectome.org/) provides the details of participants, the acquisition protocol, and preprocessing of the functional data. The data analyzed here were taken from the previous study by Fallon et al. (2020), corresponding to a subset of 100 unrelated participants (54 males, 46 females, healthy and aged between 22 and 35 years). The sequence and imaging parameters were the following: sequence, gradient-echo EPI; TR, 720 ms; TE, 33.1 ms; flip angle 52°; FOV, 208 × 180 mm (RO × PE); matrix, 104 × 90 (RO × PE); slice thickness, 2.0 mm, 72 slices, 2.0 mm isotropic voxels; multiband, factor 8; echo spacing, 0.58 ms; BW, 2,290 Hz/Px.

For details about the processing of the data, see Fallon et al. (2020). Briefly, the HCP diffusion pipeline (Glasser et al., 2013) was applied to preprocess the diffusion data, including b0 image normalization, correction for EPI susceptibility, and eddy-current–induced distortions, gradient nonlinearities, subject motion, and application of a brain mask. Tractography was performed using Fibre Assignment by Continuous Tracking (FACT; Mori et al., 1999; Mori and Van Zijl, 2002), in combination with Anatomically Constrained Tractography (ACT; Smith et al., 2012) and Spherically Informed Filtering of Tractograms (SIFT-2; Smith et al., 2015). The preprocessing of the rs-fMRI time series included white matter, mean cerebrospinal fluid, and global signal regressions, linear detrending, and high-pass filter as a hard threshold at 8 × 10^−3^ Hz. Finally, time series for all ROIs were obtained by averaging voxel time series over all voxels within each parcel.

### Parcellation and connection strength

Cortical regions of interest (ROIs) were defined using three different cortical parcellations: the first one followed the Desikan–Killiany (DK) atlas (Desikan et al., 2006; *N* = 68 ROIs, i.e., 34 regions per hemisphere); the second was the 200-node parcellation (*N* = 200 ROIs, i.e., 100 regions per hemisphere) from Fallon et al. (2020); the third was the 360-region HCP parcellation (Glasser et al., 2016; *N* = 360 ROIs, i.e., 180 regions per hemisphere). See Fallon et al. (2020) for details. The connection strength was defined as the weighted degree of the connectome, equal to $S_{j}=\sum_{k}W_{\;jk}$ for ROI *j*, where $W=\{W_{\;jk}\}$ is the connectome matrix. Connection strengths were normalized by the mean value across nodes, i.e., $S_{j}\to S_{j}/\left\langle S_{j}\right\rangle$. Additionally, the centrality of brain regions within the connectome was measured using betweenness centrality (Rubinov et al., 2009). Briefly, node betweenness centrality is defined as the fraction of shortest paths that passes through a given node. To calculate the betweenness centrality, the connectome was first normalized by its maximum value, i.e., $W_{\;jk}\to W_{\;jk}/\max(W_{\;jk})$, and then binarized with a threshold equal to 0.005.

### T1w/T2w-based heterogeneity maps

The HCP MRI dataset includes bias field-corrected maps representing the ratio between T1-weighted and T2-weighted images (T1w/T2w). These T1w/T2w maps were averaged across subjects to produce an average T1w/T2w map, which served as an estimate of regional heterogeneity (Demirtaş et al., 2019). Previous studies have shown that the T1w/T2w map is correlated with the intracortical myelin content (Glasser and Van Essen, 2011; Glasser et al., 2014), which varies along a sensory-association gradient (Margulies et al., 2016; Huntenburg et al., 2017). Moreover, the T1w/T2w map correlates with the number of spines on pyramidal cell dendrites and gene expression gradients (Burt et al., 2018). For these reasons, the T1w/T2w map has been proposed as a noninvasive marker of anatomical hierarchy in the primate cortex (Burt et al., 2018).

### Whole-brain models

Whole-brain models are composed of local nodes representing ROIs (or brain regions) that are interconnected through anatomical connections given by the connectome matrix $W=\{W_{\;jk}\}$ averaged over subjects in the DK parcellation. To get values between 0 and 1, the connectome matrix was normalized by the maximum value of its elements, i.e., $W_{\;jk}\to W_{\;jk}/\max(W_{\;jk})$. As described below, the models used here differ in the local dynamics, which can relax and/or oscillate, and the coupling functions, which can be linear, diffusive, or nonlinear. Linear stability analysis was used to study the equilibrium points of the models.

#### Linear model

In this model, the activity of the network is governed by the following system of linear stochastic differential equations (Ornstein–Uhlenbeck process):

$$\frac{d\vec{x}}{dt}=-\alpha \vec{x}+gW\vec{x}+\vec{\eta },\;\;(1)$$

where coupling matrix W is the connectome; α represents an inverse time constant (set to 1); $\vec{\eta }$ denotes an *N*-dimensional uncorrelated white noise, i.e., $\left\langle \eta_{j}(t)\right\rangle =0$ and $\left\langle \eta_{j}(t)\eta_{k}(t^{\prime })\right\rangle =\sigma^{2}\delta (t-t^{\prime })\delta_{\;jk}$; and σ is the amplitude of the noise.

#### Hopf model

The whole-brain dynamics of the Hopf model are obtained by coupling the local oscillatory dynamics of N nodes interconnected through the connectome coupling matrix W (Moon et al., 2015; Deco et al., 2017; Ponce-Alvarez and Deco, 2024). The state variables of the network are given by the system of complex-valued stochastic coupled nonlinear differential equations:

$$\frac{dz_{j}}{dt}=(a_{j}+i\omega )z_{j}-|z_{j}{|}^{2}z_{j}+g\overset{N}{\underset{k=1}{\sum }}\;W_{\;jk}(z_{k}-z_{j})+\eta_{j},\;\;(2)$$

for j∈[1,…,N], where i is the imaginary unit, g (in s^−1^) represents a global scaling of the connectivity W, and $\eta_{j}$ is the uncorrelated white noise of amplitude σ. In this model, the state variables $z_{j}$ are complex numbers whose real parts, xj=Re(zj), are assumed to represent the fMRI signals. The parameter ω represents the intrinsic angular frequencies of the nodes; it was equal to ω=2π rad·s^−1^ for all nodes. The local bifurcation parameters $a_{j}$ control the stability of isolated nodes. Here, two versions of this model were studied: the homogenous case for which the local bifurcation parameter is constant across nodes (i.e., $a_{j}=a$) and the heterogeneous case for which nodes can have different local bifurcation parameters $a_{j}$. In this later case, the heterogeneity in the values of $a_{j}$ can be either unstructured, i.e., drawn from a normal distribution $N(a_{0},\Delta a)$ with mean $a_{0}$ and standard deviation Δa=0.2, or structured by a T1w/T2w-based map of regional heterogeneity (see below). The diffusive coupling function $\left(z_{k}-z_{j}\right)$ promotes phase synchronization between coupled nodes, making the system behave as an extension of the Kuramoto model to the case in which both the phase and the amplitude of the oscillators are allowed to vary and interact (Moon et al., 2015; Ponce-Alvarez and Deco, 2024). A nondiffusive version of the interaction function replaces the term $g\sum_{k=1}^{N}W_{\;jk}(z_{k}-z_{j})$ in Equation 2 by $g\sum_{k=1}^{N}W_{\;jk}z_{k}$.

#### Wilson–Cowan model

Neural activity was simulated using a population firing rate model based on the Wilson–Cowan equations (Wilson and Cowan, 1972). Each local node consists of an excitatory and an inhibitory neuronal population, whose dynamics are governed by the following stochastic differential equations:

$$\tau_{E}\frac{dr_{j}^{\left(E\right)}}{dt}=-r_{j}^{\left(E\right)}+f(u_{j}^{\left(E\right)})+\eta_{j}^{\left(E\right)},\;\;(3)$$

$$\tau_{I}\frac{dr_{j}^{\left(I\right)}}{dt}=-r_{j}^{\left(I\right)}+f(u_{j}^{\left(I\right)})+\eta_{j}^{\left(I\right)},\;(4)$$

$$u_{j}^{\left(E\right)}=w_{EE}r_{j}^{\left(E\right)}-w_{EI}r_{j}^{\left(I\right)}+g\underset{k\neq j}{\sum }\;W_{\;jk}r_{k}^{\left(E\right)}+h_{E},\;\;(5)$$

$$u_{j}^{\left(I\right)}=w_{IE}r_{j}^{\left(E\right)}-w_{II}r_{j}^{\left(I\right)}+g\underset{k\neq j}{\sum }\;W_{\;jk}r_{k}^{\left(E\right)}+h_{I},\;(6)$$

where $r_{j}^{\left(E\right)}$ and $r_{j}^{\left(I\right)}$ represent the mean activity of the *E* and *I* neural populations of brain region j∈[1,…,N], respectively. The nonlinear transfer function f is the sigmoid function: $f(u)=[1+e^{-u}{]}^{-1}$. The variables $u_{j}^{\left(E\right)}$ and $u_{j}^{\left(I\right)}$ represent the inputs to *E* and *I* populations of brain region j, respectively. An *E* population receives a constant background input $h_{E}$, self-excitation (with strength $w_{EE}$), local inhibition (with strength $w_{EI}$), and long-range inputs from other *E* populations (with strengths given by $gW_{\;jk}$). Similarly, an *I* population receives a background input $h_{I}$, self-inhibition (with strength $w_{II}$), local excitation (with strength $w_{IE}$), and long-range inputs from other *E* populations (with strengths $gW_{\;jk}$). Note that the long-range connection from the *E* population in node j to the *I* population in node k induces inhibition to the *E* population in node k through the local connection from *I* to *E*. This is called long-range feedforward inhibition. The temporal scales of the dynamics of isolated nodes are given by the time constants $\tau_{E}$ and $\tau_{I}$, with $\tau_{E}=2\tau_{I}$ and $\tau_{E}=1$. Finally, $\eta_{j}^{\left(E\right)}$ and $\eta_{j}^{\left(I\right)}$ are uncorrelated white noises of amplitude σ. Unless otherwise stated, the local coupling strengths $w_{EE}$, $w_{EI}$, $w_{IE}$, and $w_{II}$ are fixed and equal to $w_{EE}=w_{EI}=\;12$, $w_{IE}=\;16$, and $w_{II}=\;4$. The global coupling g is also fixed and equal to 1.5× ‖W‖. The free parameters of the model are the background inputs $h_{E}$ and $h_{I}$. In structured heterogeneous models, the recurrent excitatory couplings $\left(w_{EE}\right)$ were modulated by the T1w/T2w-based map of regional heterogeneity (see below).

In vector form, the state of the network is described by an 2N-dimensional vector $\vec{r}=[r_{1}^{\left(E\right)},\ldots ,r_{N}^{\left(E\right)},\;r_{1}^{\left(I\right)},\ldots r_{N}^{\left(I\right)}]$; the time constants are contained in the 2N-by- 2N diagonal matrix $T=\left[\begin{array}{cc} \tau_{E}I & 0\\ 0 & \tau_{I}I \end{array}\right]$, where I is the N-dimensional identity matrix; and the network's inputs are described by $\vec{u}=[u_{1}^{\left(E\right)},\ldots ,u_{N}^{\left(E\right)},\;u_{1}^{\left(I\right)},\ldots u_{N}^{\left(I\right)}]=M\vec{r}+\vec{h}$, where $\vec{h}=[h_{E},\ldots ,h_{E},h_{I},\ldots ,h_{I}]$ and the 2N-by- 2N matrix M contains the local and large-scale couplings as a block matrix: $M=\left[\begin{array}{cc} M_{EE} & M_{EI}\\ M_{IE} & M_{II} \end{array}\right]$, where $M_{EE}=w_{EE}I+gW$, $M_{IE}=w_{IE}I+gW$, $M_{EI}=-w_{EI}I$, and $M_{II}=-w_{II}I$, where I is the N-dimensional identity matrix. Without noise, the system of Equations 3–6 writes $T\frac{d\vec{r}}{dt}=-\vec{r}+f(M\vec{r}+\vec{h})$. Finally, a version of the model without long-range feedforward inhibition, i.e., for which interareal connections occur only between *E* populations, can be obtained by setting $M_{IE}$ equal to: $M_{IE}=w_{IE}I$, i.e., long-range connections from *E* to *I* populations are set to zero.

### Structured heterogeneity and in-degree connectivity normalization

Previous studies have examined the effect of including regional heterogeneity in large-scale brain models using cortical gradients derived from data, such as the macroscopic gradients of gray matter myelination or of excitatory connection strength (Chaudhuri et al., 2015; Demirtaş et al., 2019; Wang, 2020; Mejías and Wang, 2022; Ding et al., 2024) or using theoretical/synthetic gradients (Cocchi et al., 2016; Gollo et al., 2017; Pang et al., 2021).

Here, following Demirtaş et al. (2019), a Tw1/Tw2-based map was used here as a proxy of the hierarchical heterogeneity of brain regions. Let $H_{\mathrm{map}}$ be the hierarchical heterogeneity map, the value for the *j*-th brain region, noted $H_{\mathrm{map},j}$, is given as follows:

$$H_{\mathrm{map},j}=\frac{\max(T_{\mathrm{map}})-T_{\mathrm{map},j}}{\max(T_{\mathrm{map}})-\min(T_{\mathrm{map}})},\;(7)$$

where $T_{\mathrm{map},j}$ is the region's T1w/Tw2 value. Consequently, the $H_{\mathrm{map}}$ map has values between 0 and 1. The $H_{\mathrm{map}}$ map negatively correlates with the connectivity strength (Spearman correlation: −0.30, *p* = 0.012, for the DK parcellation). To isolate the effect of regional heterogeneity, the connectivity matrix was normalized row-wise so that all regions had equal weighted in-degree (which is equivalent to the node strength, i.e., $S_{j}=\sum_{k}W_{\;jk}$, since the connectivity matrix is symmetric; Demirtaş et al., 2019). In the structured heterogeneous models, the equations of the j-th node were modified by making the following parameter changes: $g\to \frac{g}{S_{j}}$ (connectivity normalization); $\left\{\alpha \to \alpha +\beta H_{\mathrm{map},j}\right\}$, $\left\{a_{j}\to a_{\min}+\beta H_{\mathrm{map},j}\right\}$, and $\left\{w_{EE}\to w_{EE}+\beta H_{\mathrm{map},j}\right\}$ for the linear, Hopf, and Wilson–Cowan models, respectively.

### Linear fluctuations

All three models have phases for which deterministic dynamics settle into a stable fixed point that can be a node (for the linear model and the Wilson–Cowan model) or a spiral/focus (for the Hopf and Wilson–Cowan models). For the Hopf model, the fixed point is the origin, i.e., ${\vec{z}}^{*}=\vec{0}$; for the linear model, the fixed point is the solution of the linear system $\vec{x}=\alpha^{-1}gW\vec{x}$; for the Wilson–Cowan model, the fixed point is the solution of the nonlinear system $\vec{r}=T^{-1}f(M\vec{r}+\vec{h})$. The last two solutions can be calculated numerically. Around the stable fixed point, it is possible to treat linearly the stochastic fluctuations $\delta \vec{x}$ of the state variables. This allows to calculate the stationary network's statistics (e.g., covariances, lagged covariances, and spectral densities) analytically as follows (Bressloff, 2010; Ponce-Alvarez et al., 2015; Pfeffer et al., 2021; Ponce-Alvarez and Deco, 2024). Let ${\vec{x}}^{*}$ be the fixed point (that can be a node or a focus) and A the Jacobian matrix of the system at point ${\vec{x}}^{*}$. The stationary covariance matrix $C_{v}=\left\langle \delta \vec{x}(t)\delta \vec{x}{\left(t\right)}^{T}\right\rangle$ of the fluctuations around ${\vec{x}}^{*}$, noted $\delta \vec{x}$, is given by solving the Lyapunov algebraic equation:

$$AC_{v}+C_{v}A^{T}+Q_{n}=0,\;(8)$$

where $Q_{n}=\langle \vec{\eta }{\vec{\eta }}^{T}\rangle$ is the covariance matrix of the noise. For uncorrelated noise, $Q_{n}$ is diagonal and equal to $\sigma^{2}I$. The Lyapunov equation can be solved using the eigen-decomposition of the Jacobian matrix. The variances of the network's state variables are given by the diagonal of $C_{v}$. For the different models used here, the Jacobian matrix is equal to A=gW−αI, for the linear model; $A=\;\mathrm{diag}(\vec{a}-g\vec{S}+i\omega )+gW$, for the Hopf model, where $\vec{a}=[a_{1},\ldots ,a_{N}]$ and $\vec{S}=[S_{1},\ldots ,S_{N}]$; and $A=\;T^{-1}M{\;f}^{\prime }$, for the Wilson–Cowan model, where ${\;f}^{\prime }$ is a diagonal matrix with diagonal elements equal to the derivatives of the transfer function f evaluated at the inputs associated to the fixed point, i.e., ${\;f}^{\prime }=\mathrm{diag}[{\;f}^{\prime }({\vec{u}}^{*})]$.

Moreover, the stationary lagged covariances of the state variables, defined as $C_{v}(\tau )=\left\langle \delta \vec{x}(t+\tau )\delta \vec{x}{\left(t\right)}^{T}\right\rangle$, is given as follows:

$$C_{v}(\tau )=e^{\tau A}\left\langle \delta \vec{x}(t)\delta \vec{x}{\left(t\right)}^{T}\right\rangle =e^{\tau A}C_{v}(0),\;(9)$$

where $C_{v}(0)=C_{v}$ is the covariance matrix (i.e., zero-lag). ACFs correspond to the normalized lagged-autocovariances, i.e., the diagonal elements of $C_{v}(\tau )$ normalized by the diagonal elements of $C_{v}(0)$.

Finally, using the Fourier transform F, it can be shown that, at each frequency ν, the power spectral densities (PSDs) of the nodes, $\phi_{j}(\nu )=\left\langle {\left|F(\delta \vec{x})\right|}^{2}\right\rangle$, are given as follows:

$$\phi_{j}(\nu )=\sigma^{2}\underset{k}{\sum }\;|{\left(A_{\;jk}+i2\pi \nu \right)}^{-1}{|}^{2}.\;(10)$$

### Autocorrelation function decay

For each ROI in the data, the autocorrelation function (ACF), noted C(τ), was calculated. The decay length of the ACF, noted ξ, was defined as the time lag τ from which C(τ)<0.1, after linear interpolation. For the models, two calculations of ξ were used, depending on the presence or absence of oscillations. For the linear model and the Wilson–Cowan model in the stable fixed point regime, for which oscillations are absent, the ACF was an exponentially decaying function and ξ was defined as the exponential decay constant, i.e., $C(\tau )=e^{-\tau /\xi }$. For the Hopf model and the Wilson–Cowan model in the stable spiral regime, for which damped oscillations are observed, the envelop of the ACF, noted env[C(τ)], was an exponentially decaying function with decay constant equal to ξ, i.e., $\mathrm{env}[C(\tau )]∼e^{-\tau /\xi }$.

### Network stimulation

The response of the brain network was tested using the homogeneous Hopf model for which an oscillatory input of amplitude $A_{\mathrm{in}}$ and angular frequency ω was applied separately to each node. Let l be the stimulated node; the system's equations became the following:

$$\frac{dz_{j}}{dt}=(a+i\omega )z_{j}-|z_{j}{|}^{2}z_{j}+g\overset{N}{\underset{k=1}{\sum }}\;W_{\;jk}(z_{k}-z_{j})+\delta_{\;jl}A_{\mathrm{in}}e^{i\omega t}+\eta_{j},\;(11)$$

where $\delta_{\;jl}$ is the Kronecker delta and j∈[1,…,N]. The model was homogeneous with a=−0.1 and g=15×‖W‖. The stimulus had the same angular frequency as the nodes, i.e., ω=2π rad·s^−1^, and its amplitude $A_{\mathrm{in}}$ varied from 0.1 to 10. For each stimulus amplitude and for each stimulated node, the stochastic system was simulated for T=40s and the stationary regime was assumed to be reached after 30 s. The response of the nodes was given by the average modulus of its state variable during the stationary response, i.e., $r_{j}=\left\langle \left|z_{j}\right|\right\rangle$, for 1≤j≤N. The response of the nodes was studied as a function of the stimulus amplitude, i.e., $r_{j}(A_{\mathrm{in}})$. The susceptibility of the stimulated node, $\chi_{l}$, was given by the sum of the response over tested stimulus amplitudes, i.e., $\chi_{l}=\sum_{\left\{A_{\mathrm{in}}\right\}}r_{l}(A_{\mathrm{in}})$. The propagation susceptibility, $\chi_{\mathrm{prop}}$, was given by the sum of the average response of the nonstimulated nodes over stimulus amplitudes, i.e., $\chi_{\mathrm{prop}}=\sum_{\left\{A_{\mathrm{in}}\right\}}\frac{1}{N-1}\sum_{\;j\neq l}r_{j}(A_{\mathrm{in}})$.

### Statistical analyses

Unless otherwise specified, all reported correlations used Pearson’s correlation. When tests were repeated across single-subject data, *p* values were corrected using the false discovery rate. The specific statistical test applied is noted alongside the results. Curve fittings were performed using nonlinear least-squares. Statistical tests and fittings were conducted in MATLAB using built-in functions. The significance threshold was set at p<0.05.

### Reanalysis of published data

The data subset used here was the same from Fallon et al. (2020). In that study, the timescales of fMRI signals were calculated using the relative low-frequency power. Here, timescales were estimated using the decay of the autocorrelation function with time lag, as described above. This measure has been previously used to quantify the timescale of fMRI signals (Murray et al., 2014; Watanabe et al., 2019).

### Materials availability

The present work used a publicly available dMRI and fMRI data from the Human Connectome Project (HCP). The HCP dataset is available at https://www.humanconnectome.org/study/hcp-young-adult.

The data subset used here was the same from Fallon et al. (2020) and it is available here: https://github.com/NeuralSystemsAndSignals/humanStructureFunction.

### Code availability

The codes to perform the numerical simulations and to estimate the network statistics using the linear approximation for the three models used here are available at https://github.com/adrianponce/BrainScales. In this repository, the structural connectivity matrices and the regional heterogeneity map used here were also included.

## Results

### Structure‒function relationships linking the variance and the temporal scales of resting-state activity and brain connectivity

The present work uses publicly available data from the Human Connectome Project. The dataset used here is the one used in Fallon et al. (2020), which consists of connectome matrices and resting-state (rs) fMRI signals from 100 subjects. Cortical ROIs were defined using three different parcellations: the first one followed the Desikan–Killiany (DK) atlas (Desikan et al., 2006; *N* = 68 ROIs); the second was the 200-node parcellation (*N* = 200 ROIs) from Fallon et al. (2020); the third was the 360-region HCP parcellation (Glasser et al., 2016; *N* = 360 ROIs).

**Figure 1.**
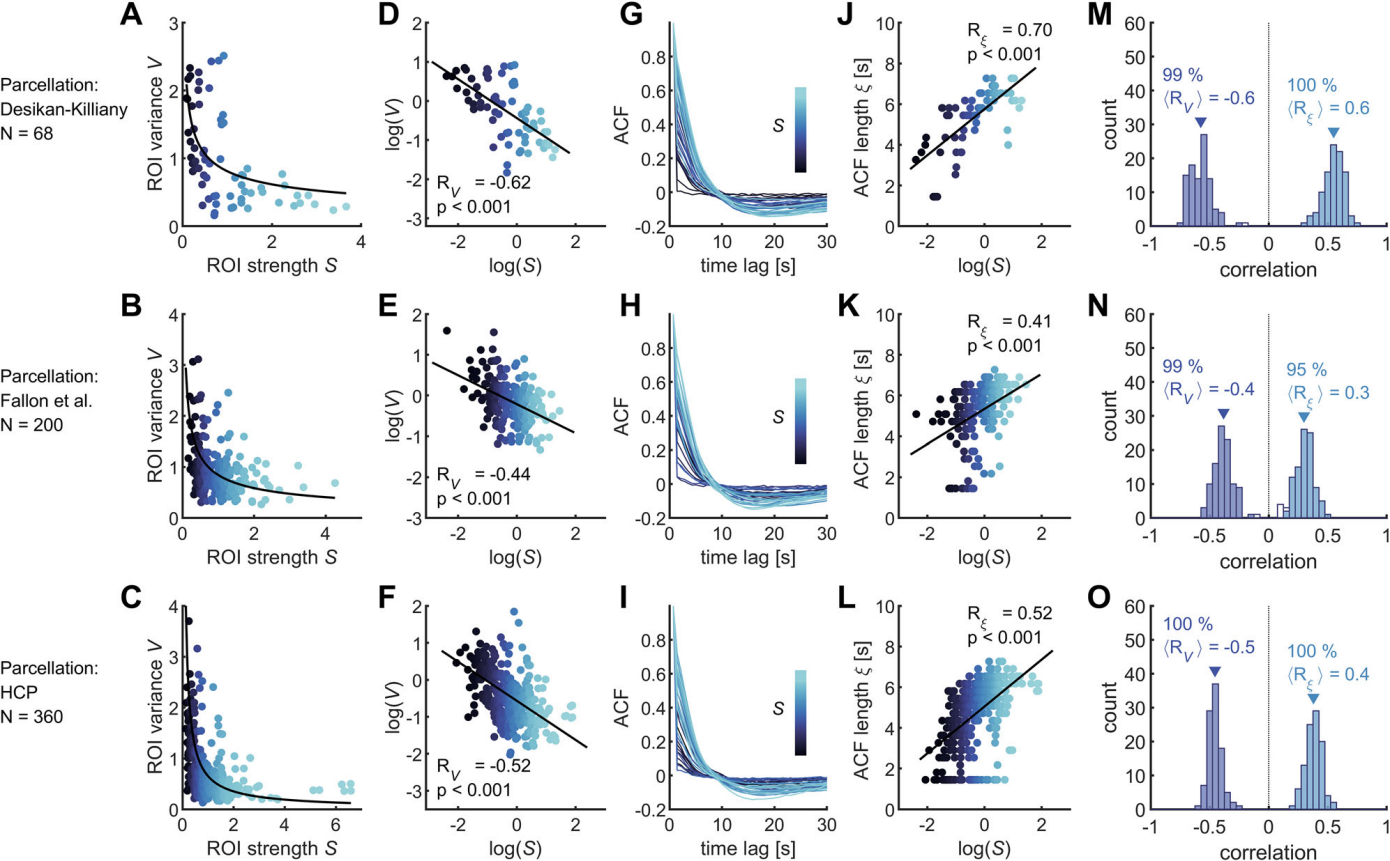
Relation between brain connectivity and the variance and temporal scales of rs-fMRI. ***A–C***, Relation between the variance V of the rs-fMRI signals of the ROIs and their corresponding structural connectivity strength S, for the DK parcellation (***A***), the Fallon parcellation (***B***), and the HCP parcellation (***C***). Solid lines indicate power law fits. ***D–F***, Same as ***A–C*** but in log-log plot. The coefficients $R_{V}$ and the *p* values of the correlation between log(S) and log(V) are indicated for the three different parcellations. ***G–I***, Autocorrelation function (ACF) of rs-fMRI signals, for the three different parcellations. The ACF decays were longer for nodes with larger connection strength. ***J–L***, The ACF length ξ (time lag for which the ACF became < 0.1) positively correlated with the ROI's strength S. The coefficients $R_{\xi }$ and the *p* values of the correlation between log(S) and ξ are indicated for the three different parcellations. ***M–O***, Histograms of correlation coefficients between log(S) and log(V) and between log(S) and ξ for single-subject data (*n* = 100 subjects), for the DK parcellation. Nonsignificant correlation coefficients (after false discovery rate correction of the *p* values) are indicated in white. The percentage of significant correlations and the average correlation coefficient are also indicated.

**Figure 2.**
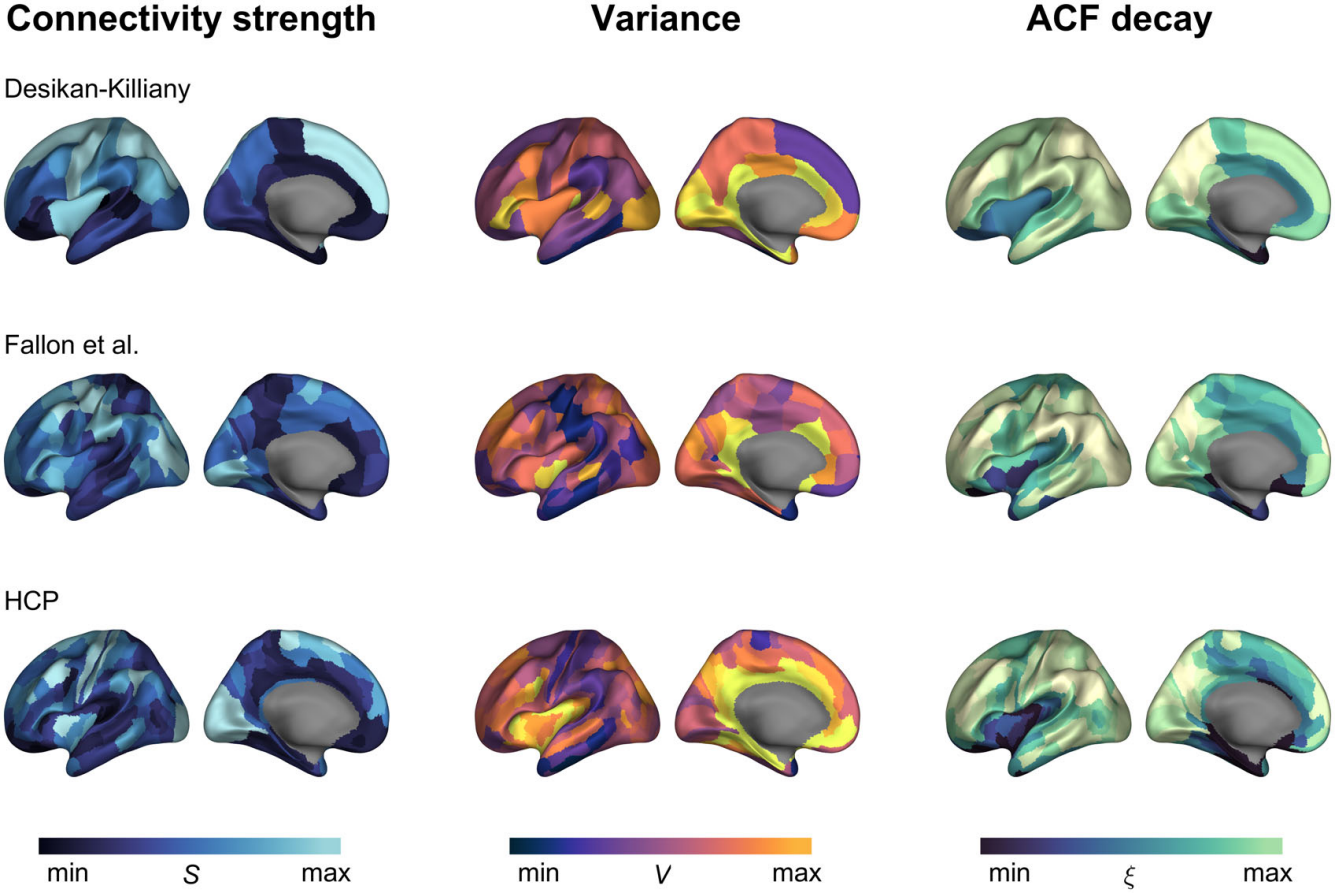
Spatial maps of connectivity, variance, and timescale. Spatial maps of structural connectivity strength (first column), signal variance (second column), and ACF decay (third column) for the brain regions in the left-hemisphere cortical, for the three parcellations (rows). The maps were averaged over subjects.

For each parcellation and each ROI, the variance of the rs-fMRI signal V, the node's strength of the connectome S, and the autocorrelation function (ACF) were calculated and averaged over subjects. For the three parcellations, the variance of the activity of the ROIs decreased as a function of their connection strength (Fig. 1*A–C*). Indeed, the correlation between logS and logV, noted $R_{V}$, was significant and negative: *R_v_ *= −0.62 (*p* < 0.001), −0.44 (*p* < 0.001), and −0.52 (*p* < 0.001), for DK, Fallon et al., and HCP parcellations, respectively (Fig. 1*D–F*). Moreover, consistent with previous findings (Murray et al., 2014; Fallon et al., 2020), the ACF presented a slower decay for strongly connected ROIs (Fig. 1*G–I*). The length of the ACF, noted ξ, was defined as the time lag from which ACF < 0.1. The correlation between logS and ξ, noted $R_{\xi }$, was significant and positive: $R_{\xi }=\;0.70$ (*p* < 0.001), 0.41 (*p* < 0.001), 0.52 (*p* < 0.001), for DK, Fallon et al., and HCP parcellations, respectively (Fig. 1*J–L*). Therefore, the structural connectivity correlated negatively with the magnitude of activity fluctuations (i.e., the variance) and positively with the temporal scale (i.e., the ACF length). Furthermore, these results were consistently found using single-subject data (Fig. 1*M–O*). Finally, the spatial maps of connectivity, variance, and timescale are shown for the three parcellations in Figure 2.

### Linear prediction of structure‒function relationships

**Figure 3.**
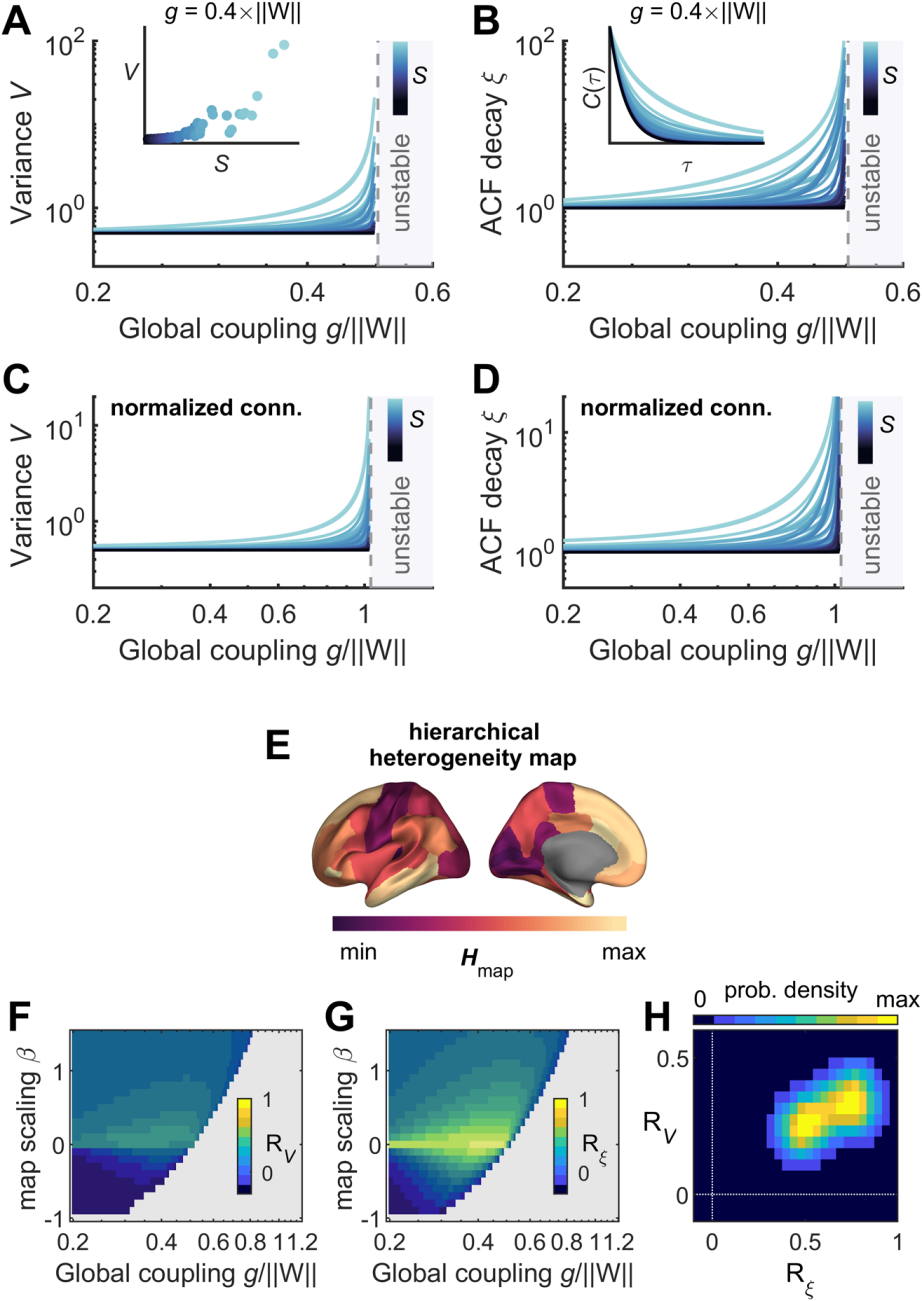
Linear prediction of the variance and temporal scales and their relationship with the brain connectivity. ***A***, Nodes’ variances V as a function of the global coupling parameter g (normalized by ‖W‖) for the linear model. The colors correspond to the associated node connection strength S. Inset, one example of variance–strength relationship for a given value of g. ***B***, Nodes’ ACF lengths ξ as a function of the global coupling parameter g. Inset, one example of ξ–strength relationship for a given value of g. ***C***, ***D***, Same as panels ***A*** and ***B*** but after normalization of the strength of connections, so that all nodes have equal in-degree. ***E***, Hierarchical regional heterogeneity map estimated using the T1w/T2w map in the DK parcellation. ***F***, ***G***, Correlation between V and S (noted $R_{V}$; panel ***F***) and between ξ and S (noted $R_{\xi }$; panel ***G***) in the parameter space of the structured heterogeneous linear model. The parameter space was defined by the global coupling parameter g and the map scaling parameter β. ***H***, Resulting joint distribution of the significant (*p* < 0.05) correlations $R_{\xi }$ and $R_{V}$, for the structured heterogeneous linear model. In panels ***A–D***, ***F***, and ***G***, the gray region corresponds to unstable dynamics.

The above results prompt the question of whether the variance and the ACF length are trivially related. A linear model was used to test whether the above results could be explained by simple network propagation principles. In this basic model, the activity of the nodes is described by a *N*-dimensional Ornstein–Uhlenbeck process with coupling matrix equal to gW, where the *N*-by-*N* matrix W is given by the averaged connectome in the DK parcellation and g is a global scaling parameter (see Materials and Methods). Briefly, in this model, the activity of the network was governed by the following linear stochastic differential equation: $d\vec{x}/dt=-\alpha \vec{x}+gW\vec{x}+\vec{\eta }$, where α represents an inverse time constant and $\vec{\eta }$ denotes an *N*-dimensional uncorrelated white noise with amplitude equal to σ. Note that the noise amplitude σ and the time constant, equal to $\alpha^{-1}$, were the same for all nodes, and, therefore, differences on amplitude and temporal scales were determined only by the network connectivity. As g increases the system loses stability until it becomes unstable. In the region of stability, the variances and the ACFs can be analytically estimated (see Materials and Methods). As the system destabilizes both the variance and the ACF length increase, and they both correlate positively with the connection strength (Fig. 3*A*,*B*). A similar behavior is observed after normalizing the connectivity matrix so that all nodes have the same in-degree, thereby removing the heterogeneity due to connectivity (Fig. 3*C*,*D*; see Materials and Methods). Next, the effect of adding structured regional heterogeneity was examined by using the T1w/T2w map from the MRI data to construct a proxy for hierarchical regional heterogeneity (see Materials and Methods), as this measure has been shown to correlate with anatomical hierarchy in the primate cortex (Burt et al., 2018). The resulting hierarchical regional heterogeneity map, $H_{\mathrm{map}}$ (Fig. 3*E*), was used to modulate the intrinsic dynamics of the nodes by changing their inverse time constants as follows: $\alpha \to \alpha +\beta H_{\mathrm{map},j}$, where β is a scaling parameter. This led to a linear model incorporating a hierarchical regional heterogeneity map, while keeping the connectivity normalized. This model, however, is unable to produce significant $R_{V}$ and $R_{\xi }$ correlations with opposing signs (Fig. 3*F–H*). In conclusion, the observed opposite correlations between variance and connection strength, and between ACF length and connection strength, cannot be trivially explained by linear dynamics, even in the case of structured heterogeneity.

### Structure‒function relationships in whole-brain models

To explore mechanisms that could account for the opposite behavior of the variance and temporal scales of resting-state fluctuations, two commonly used whole-brain models were analyzed here. These were the Stuart–Landau network, also known as Hopf whole-brain model, and the Wilson–Cowan model.

The Hopf whole-brain model is a network of nonlinear oscillators corresponding to the normal form of a supercritical Hopf bifurcation (see Materials and Methods). This model is a canonical model to study systems of coupled oscillators for which both the phase and the amplitude interact (Matthews and Strogatz, 1990). It has been used to link brain structure and dynamics in different brain states (Moon et al., 2015; Deco et al., 2017; Jobst et al., 2017; Kim et al., 2018; López-González et al., 2021; Ponce-Alvarez and Deco, 2024). Here, the couplings between the oscillators were given by the connectome in the DK parcellation averaged over subjects, noted W. The model has local parameters $a_{j}$ for each node j (that can be homogeneous, i.e., $a_{j}=a$, or heterogeneous by drawing the parameters from a normal distribution with mean $a_{0}$ and standard deviation Δa, i.e., $a_{j}\in N(a_{0},\Delta a)$) and a global scaling parameter g that scales W (see Materials and Methods). The model can have a stable equilibrium point, the origin, displaying noise-driven oscillations. In the following, the stationary variances and ACFs of the model were studied using a linear noise approximation around the stable origin (see Materials and Methods). Since the ACFs of the model are oscillatory, the ACF length was defined as the decay of the ACF's envelop, i.e., ξ is defined as the time lag from which env[ACF] < 0.1.

**Figure 4.**
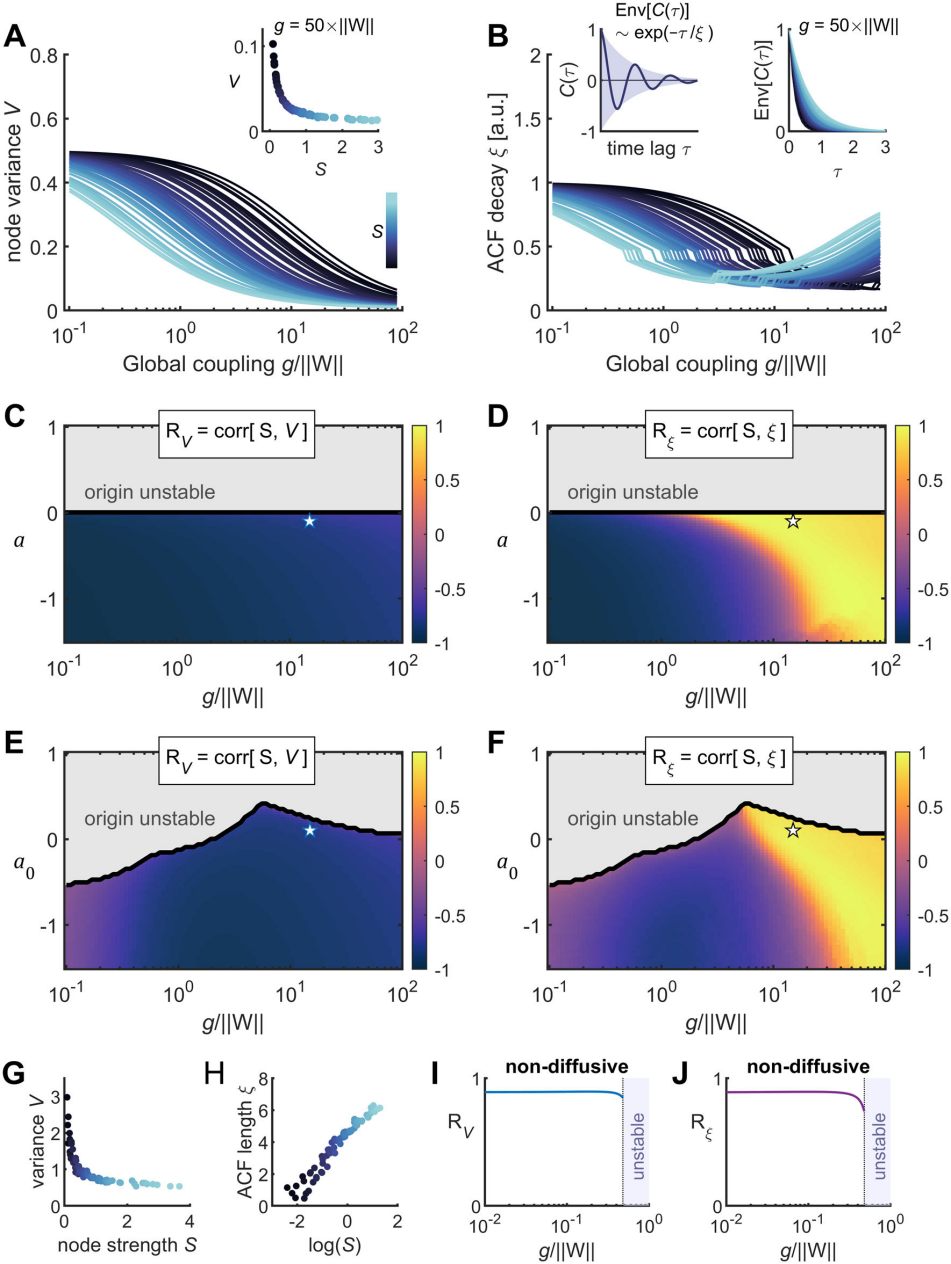
Relation between brain connectivity and the variance and temporal scales of Hopf model's fluctuations. ***A***, Linear noise approximation of nodes’ variances V as a function of the global coupling parameter g (normalized by ‖W‖); the colors correspond to the associated node connection strength S. Inset, example of variance–strength relationship for a particular value of g. ***B***, Linear noise approximation of nodes’ ACF lengths ξ as a function of the global coupling parameter g. Insets: left, the autocorrelation function (ACF) C(τ) of the model's noisy oscillations was periodic and had an exponentially decreasing envelop, i.e., Env[C(τ)]∼exp(−τ/ξ), and the length of the exponential decay, ξ, was used to quantify the temporal scale of the model's fluctuations; right, example of ξ–strength relationship for a particular value of g. ***C***, ***D***, The correlation between V and S (noted $R_{V}$; panel ***C***) and between ξ and S (noted $R_{\xi }$; panel ***D***) in the model's parameter space. In panels ***A–D***, the Hopf model was homogeneous (i.e., $a_{j}=a$ for all j∈[1,…,N]). The gray region corresponds to model parameters for which the origin is unstable, preventing the linear noise approximation. ***E***, ***F***, $R_{V}$, and $R_{\xi }$ in the parameter space of the heterogeneous Hopf model (for which $a_{j}\in N(a_{0},\Delta a)$, with Δa=0.2). ***G***, ***H***, Structure–function relationships resulting from stochastic simulations of the nonlinear heterogeneous Hopf model, for parameters located close to the origin's instability as indicated by the stars in panels ***E*** and ***F***. ***I***, ***J***, $R_{V}$, and $R_{\xi }$ for the homogeneous Hopf model (a=−1) with nondiffusive couplings. $R_{V}$ and $R_{\xi }$ were obtained using linear noise approximation. Note that $R_{V}>0$ and $R_{\xi }>0$, for all g for which the origin is stable.

In the homogeneous case, for increasing values of g, the variance V remains a decreasing function of S (Fig. 4*A*), but the relation between S and the length ξ of the ACF's envelop reverses from negative to positive for sufficiently strong coupling, i.e., g≫‖W‖, where ‖W‖ is the 2-norm of matrix W (Fig. 4*B*). The exact value of g for which such reversing occurs depends on the value of the local parameter (Fig. 4*C*,*D*). Reversing of the S−ξ correlation is also observed in the heterogeneous case that introduces a quenched disorder in the system through random differences in the intrinsic dynamics of the nodes (Fig. 4*E*,*F*). Hence, there exists a region of model parameters for which more connected nodes present lower variance and longer ACF decay in both homogeneous and heterogeneous models. These results were obtained using the linear noise approximation, but stochastic simulations of the system corroborate the results, even close to the origin's destabilization where nonlinearities are important (Fig. 4*G*,*H*). Finally, a Hopf model with nondiffusive coupling (see Materials and Methods) is not able to produce negative correlations between the connectivity strength and the variance or the ACF length (Fig. 4*I*,*J*). Thus, diffusive coupling, which promotes synchronization, is a key ingredient of the model to produce the opposite structure–function relationships.

**Figure 5.**
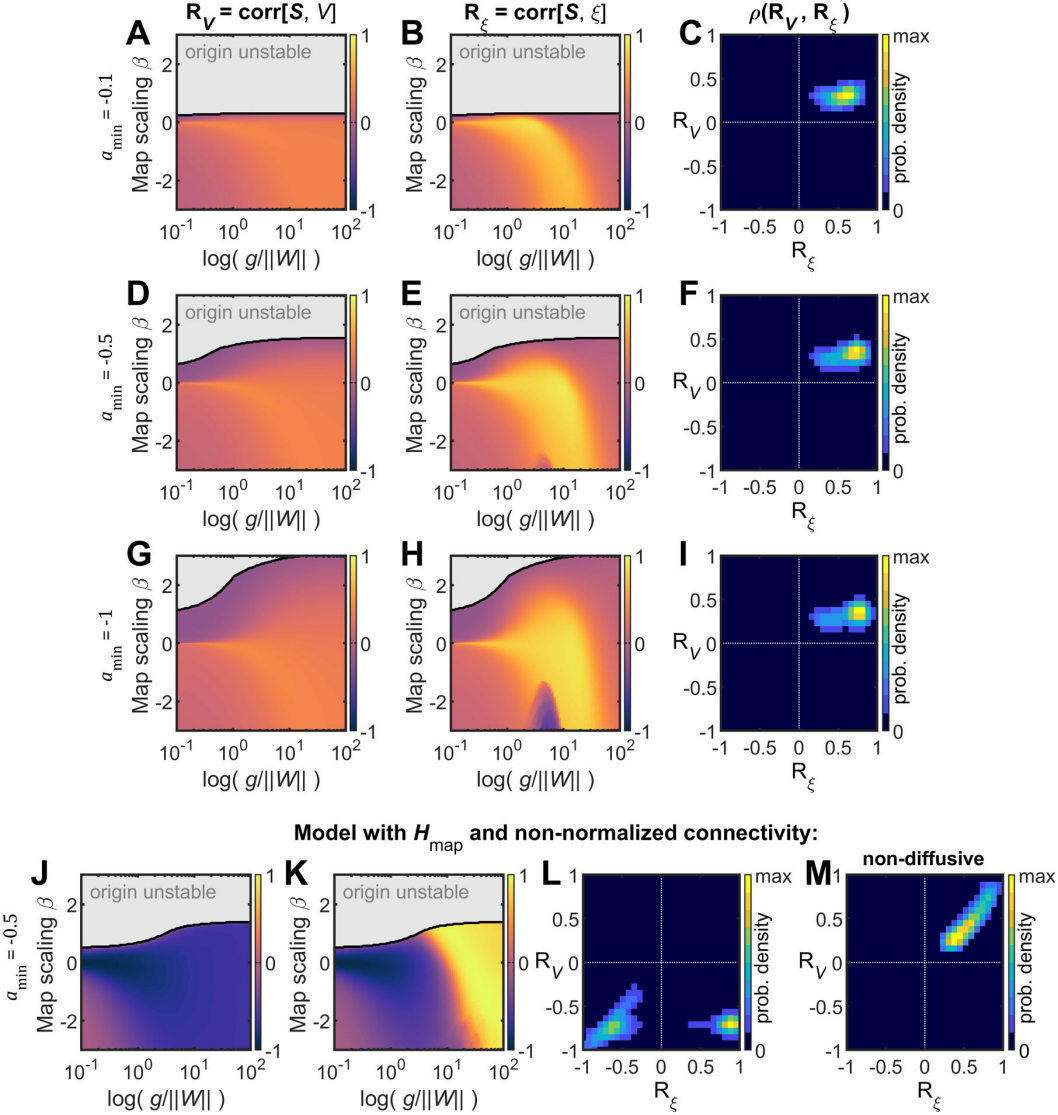
Hopf model with hierarchical regional heterogeneity map. The intrinsic dynamics of the nodes of the Hopf model were modulated by a structured regional heterogeneity map, based on T1w/T2w data, as $a_{j}=a_{\min}+\beta H_{\mathrm{map},j}$. ***A–C***, The correlations $R_{V}$ (***A***) and $R_{\xi }$ (***B***) were calculated in the (β,g) parameter space and the joint distribution of significant (p<0.05) $R_{V}$ and $R_{\xi }$ correlations was examined (***C***), for $a_{\min}=-0.1$. ***D–F***, Same as ***A–C*** but for $a_{\min}=-0.5$. ***G–I***, Same as ***A–C*** but for $a_{\min}=-1$. In panels ***A–I***, the strength of connections was normalized so that all nodes have equal in-degree. ***J–L***, Same as ***A–C*** but without normalizing the strength of connections. Note the appearance of significant $R_{V}$ and $R_{\xi }$ correlations with opposing signs (***L***). ***M***, When the connectivity is not normalized and the couplings are nondiffusive, $R_{V}$ and $R_{\xi }$ become positive.

Next, these results were compared with those obtained with a Hopf model in which the intrinsic dynamics of the brain regions were modulated by a regional heterogeneity map, while heterogeneity due to interareal connectivity was suppressed through connectivity normalization (see Materials and Methods). This version of the model introduces a parameter β that scales the regional heterogeneity map $H_{\mathrm{map}}$ (see Materials and Methods). In this model, $R_{V}$ and $R_{\xi }$ correlations can be either positive or negative within the parameter space; however, jointly significant (*p* < 0.05) correlations cannot have opposing signs (Fig. 5*A–I*). Relaxing the normalization constraint on the connectivity results in a model where heterogeneity is determined by both the hierarchical map, which affects the local parameters, and the diversity of connection strengths. In this latter model, significant $R_{V}$ and $R_{\xi }$ correlations can have opposing signs, i.e., $R_{V}<0$ and $R_{\xi }>0$ (Fig. 5*J–L*), indicating that diverse connection strengths are essential for the model to explain the structure–function relationships observed in the data. Finally, when the connectivity is not normalized and the couplings are nondiffusive, $R_{V}$ and $R_{\xi }$ become positive (Fig. 5*M*).

The Hopf model is a canonical model for capturing the synchronization of oscillators through phase and amplitude interactions, but it does not explicitly account for the neuronal or synaptic mechanisms that drive large-scale brain synchronization. Noisy oscillations around a fixed point can be generated by more realistic yet simple models like the Wilson–Cowan model, which includes interconnected excitatory (*E*) and inhibitory (*I*) neural populations, offering a closer approximation to underlying neural dynamics. Indeed, both models exhibit a supercritical Hopf bifurcation—in fact the Stuart–Landau model in polar coordinates is the normal form for this bifurcation (Ponce-Alvarez and Deco, 2024).

**Figure 6.**
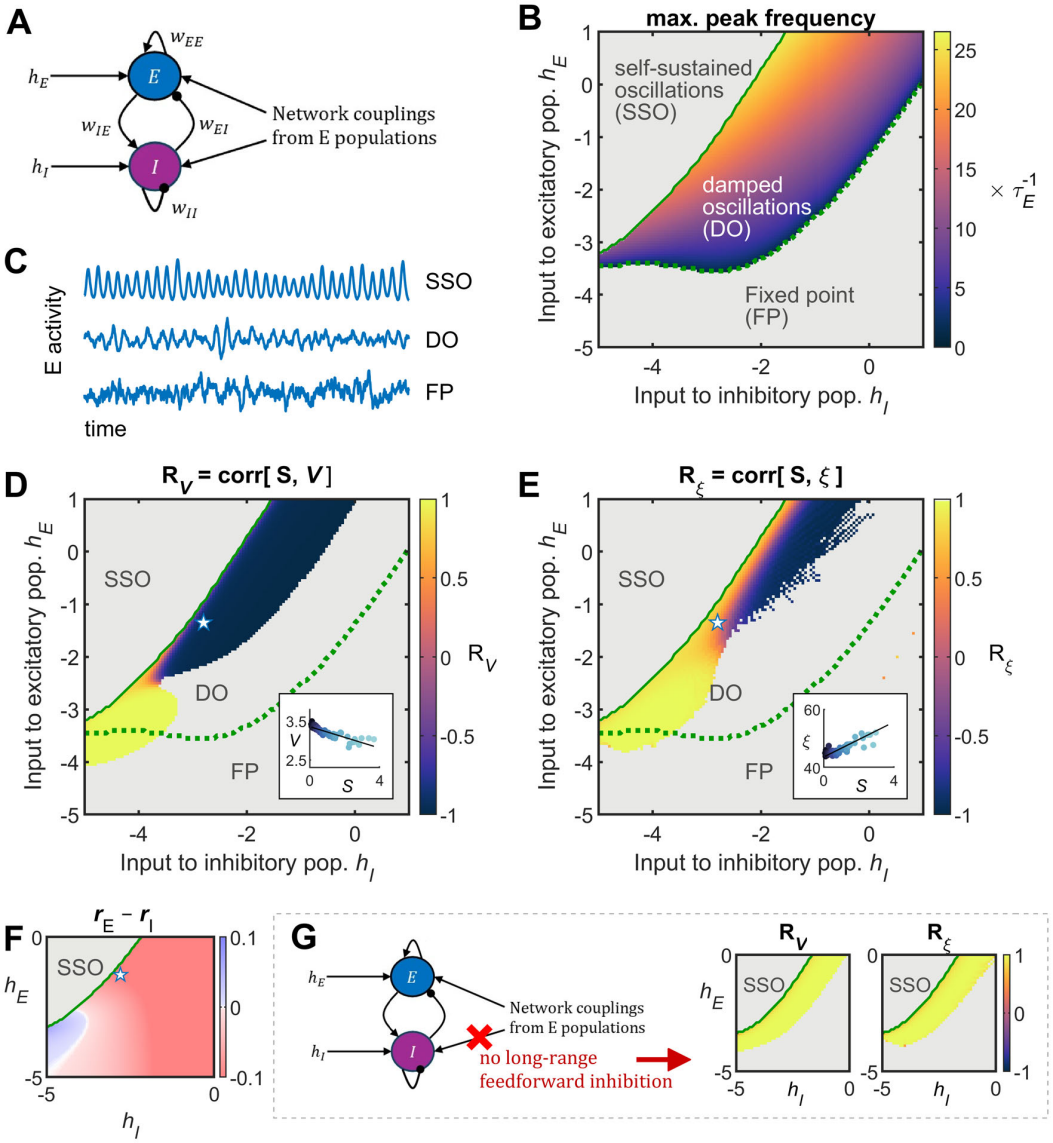
Structure–function relationships of the Wilson–Cowan model. ***A***, Model's architecture: a node is composed of interconnected excitatory (*E*) and inhibitory (*I*) neuronal populations that both receive inputs from the *E* populations of other nodes in the network as given by the connectome couplings. In addition, *E* and *I* populations receive background inputs, noted $h_{E}$ and $h_{I}$, respectively, which are the free parameters of the model. ***B***, Phase diagram, depending on the parameters $h_{E}$ and $h_{I}$, the network can display self-sustained oscillations (SSO), damped oscillations (DO), or a fixed point (FP) with no oscillations. Using linear noise approximation, the network's covariances, ACFs, and spectral densities can be estimated in the FP and DO regimes. In the DO regime, the spectral densities present a peak at nonzero frequencies and the maximum peak frequency increases close to the bifurcation (solid green line) above which SSO appear. ***C***, Examples of *E* stochastic activity for each regime; $h_{I}=\;-2.5$ and $h_{E}=\;-3.8$ (FP), −1 (DO), or 0.5 (SSO). ***D***, Correlation between V and S $\left(R_{V}\right)$. The correlation is shown for regions in the parameter space for which ΔV>5%. ***E***, Correlation between ξ and S $\left(R_{\xi }\right)$, shown for regions in the parameter space for which Δξ>5%. In panels ***D*** and ***E***, the stars indicate the parameters used to simulate the stochastic nonlinear system, for which the S−V and S−ξ relationships are shown in the insets. ***F***, Difference between the network's average excitatory activity minus the average inhibitory activity. ***G***, Left, Version of the model without long-range connections from ***E*** to ***I*** populations. Middle, $R_{V}$ in the $\left(h_{I},h_{E}\right)$ parameter space of this alternative model. Right, $R_{\xi }$ parameter space of this alternative model.

In the Wilson–Cowan model, a node is composed of a local network with *E* and *I* populations that both receive inputs from the *E* populations of other nodes in the network as given by the connectome couplings (Fig. 6*A*; see Materials and Methods). In addition, *E* and *I* populations receive background inputs, noted $h_{E}$ and $h_{I}$, respectively, which are the free parameters of the model. Depending on the parameters $h_{E}$ and $h_{I}$ the network can display self-sustained oscillations (SSO), damped oscillations (DO), or a fixed point (FP) with no oscillations (Fig. 6*B*,*C*). The injected noise produces stochastic oscillations in the DO regime. The SSO is reached after crossing a Hopf bifurcation separating the DO and the SSO regimes. In the SSO phase, dynamics are strongly nonlinear and the network oscillates autonomously. The present study concentrates on the DO and FP regimes for which the network activity mimics the noisy activity usually observed in the cortex. In these regimes the linear noise approximation can be used to estimate the network's statistics, such as the variances and ACFs of the *E* populations in the parameter space.

The resulting relationship between the nodes’ variances and their connectivity strengths and between the nodes’ ACF decays and their connectivity strengths was explored in the $\left(h_{E},h_{I}\right)$ parameter space. In this model, not all combinations of the parameters $h_{E}$ and $h_{I}$ yield differences in variances V and the decay of ACF envelops ξ for different nodes. To quantify the variations of V and ξ, the relative range of variation, given as $\Delta V=(V_{\max}-V_{\min})/V_{\min}$ and $\Delta \xi =(\xi_{\max}-\xi_{\min})/\xi_{\min}$, were calculated. It is here assumed that detectable variations of V and ξ require corresponding relative range of variations larger than 5%. Exploration of the $\left(h_{E},h_{I}\right)$ parameter space shows that, close to the Hopf bifurcation, variances differ between nodes (Δ*V* > 5%) and the correlation between nodes’ variances V and connection strengths S $\left(R_{V}\right)$ can be either negative or positive for low or high background inputs, respectively (Fig. 6*D*). Within that parameter region, the ACF lengths ξ also differ between nodes and the correlation between ξ and S $\left(R_{\xi }\right)$ changes sign for different background inputs (Fig. 6*E*). Particularly, there exist a region of parameters for which one can observe a negative V−S correlation and a positive ξ−S correlation, as well of noisy oscillations. Next, because this parameter region lies in the vicinity of the Hopf bifurcation where nonlinearities become important, these results were verified using stochastic simulations of the nonlinear system (Fig. 6*D*,*E*, insets). Note also that in this parameter region inhibition balances or dominates the excitation (Fig. 6*F*). Finally, a model without long-range feedforward inhibition, i.e., a model for which interareal connections occur only between excitatory populations (see Materials and Methods), is not able to produce negative correlations between the connectivity strength and the variance or the ACF length (Fig. 6*G*).

**Figure 7.**
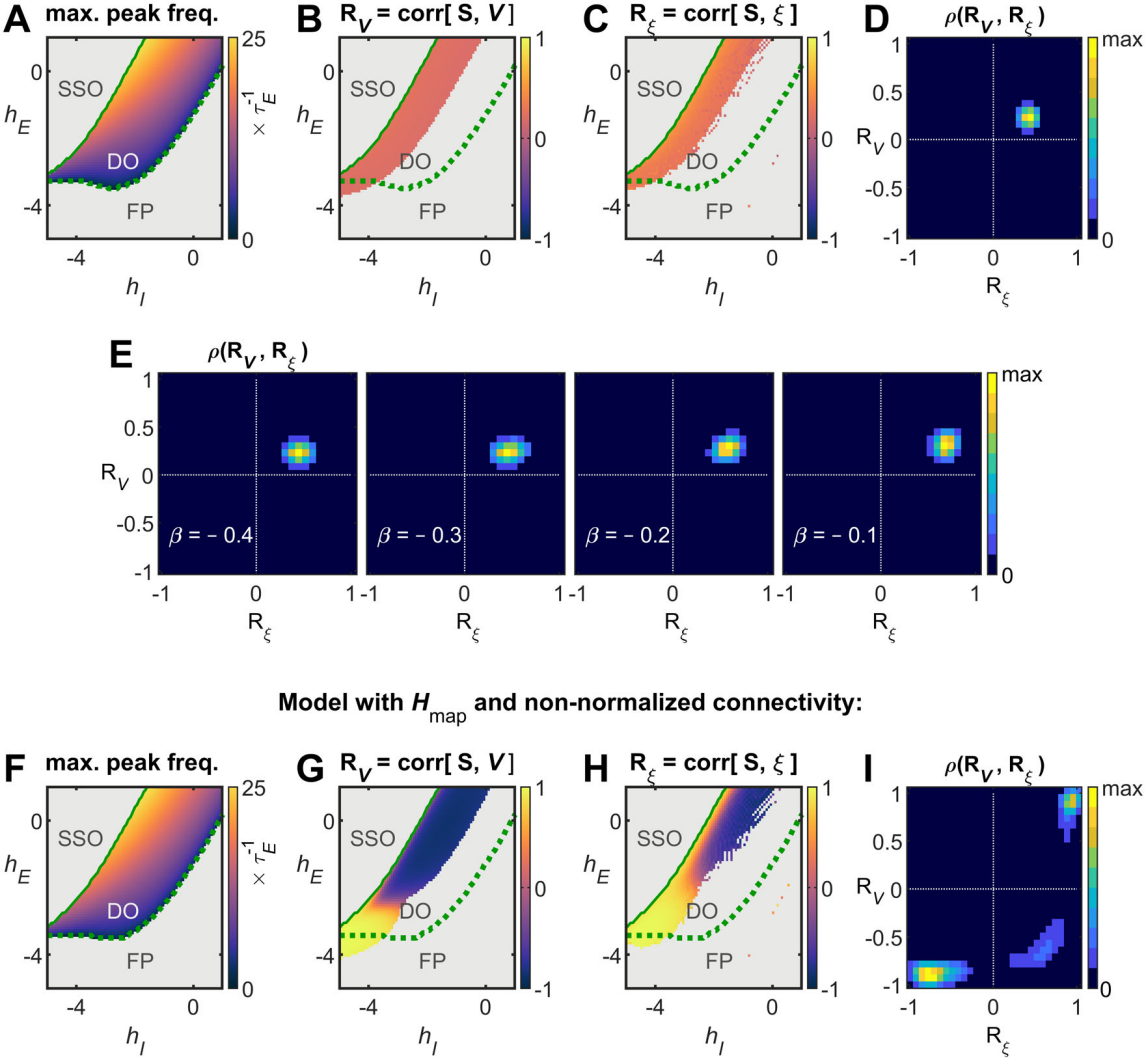
Wilson–Cowan model with hierarchical regional heterogeneity map. The recurrent excitation of the Wilson–Cowan nodes was modulated by the T1w/T2w-based regional heterogeneity map, as $w_{EE}\to w_{EE}+\beta H_{\mathrm{map},j}$. ***A–D***, In the $\left(h_{I},h_{E}\right)$ parameter space (***A***), the correlations $R_{V}$ (***B***) and $R_{\xi }$ (***C***) were calculated and the joint distribution of significant (p<0.05) $R_{V}$ and $R_{\xi }$ correlations was examined (***D***), for β=−0.5. ***E***, Joint distribution of significant (p<0.05) $R_{V}$ and $R_{\xi }$ correlations, for different values of β. ***F–I***, Same as ***A–D***, but without normalizing the strength of connections. Note the appearance of significant $R_{V}$ and $R_{\xi }$ correlations with opposing signs (***I***).

Next, these results were compared with those obtained with a Wilson–Cowan model that incorporates a T1w/T2w-based map of regional heterogeneity. It has been shown that the T1w/T2w map correlates with the number of spines on pyramidal cell dendrites, which has been related to the strength of recurrent excitation (Elston, 2003, 2007; Chaudhuri et al., 2015). For this reason, the excitatory-to-excitatory coupling strength $\left(w_{EE}\right)$ was modulated by the regional heterogeneity map $H_{\mathrm{map}}$, which was scaled by a parameter β (see Materials and Methods). Heterogeneity due to interareal connectivity was suppressed through connectivity normalization. In this model, for β<0, jointly significant (*p* < 0.05) correlations $R_{V}$ and $R_{\xi }$ are both positive (Fig. 7*A–E*), while for β>0, the correlations $R_{V}$ and $R_{\xi }$ are not jointly significant. As for the Hopf model, relaxing the normalization constraint on the connectivity leads to significant $R_{V}$ and $R_{\xi }$ correlations that can have opposing signs, i.e., $R_{V}<0$ and $R_{\xi }>0$ (Fig. 7*F–I*), thus confirming the importance of the connectivity-driven heterogeneity to explain the observed structure–function relationships.

### Susceptibility to external stimuli and core-periphery structure

**Figure 8.**
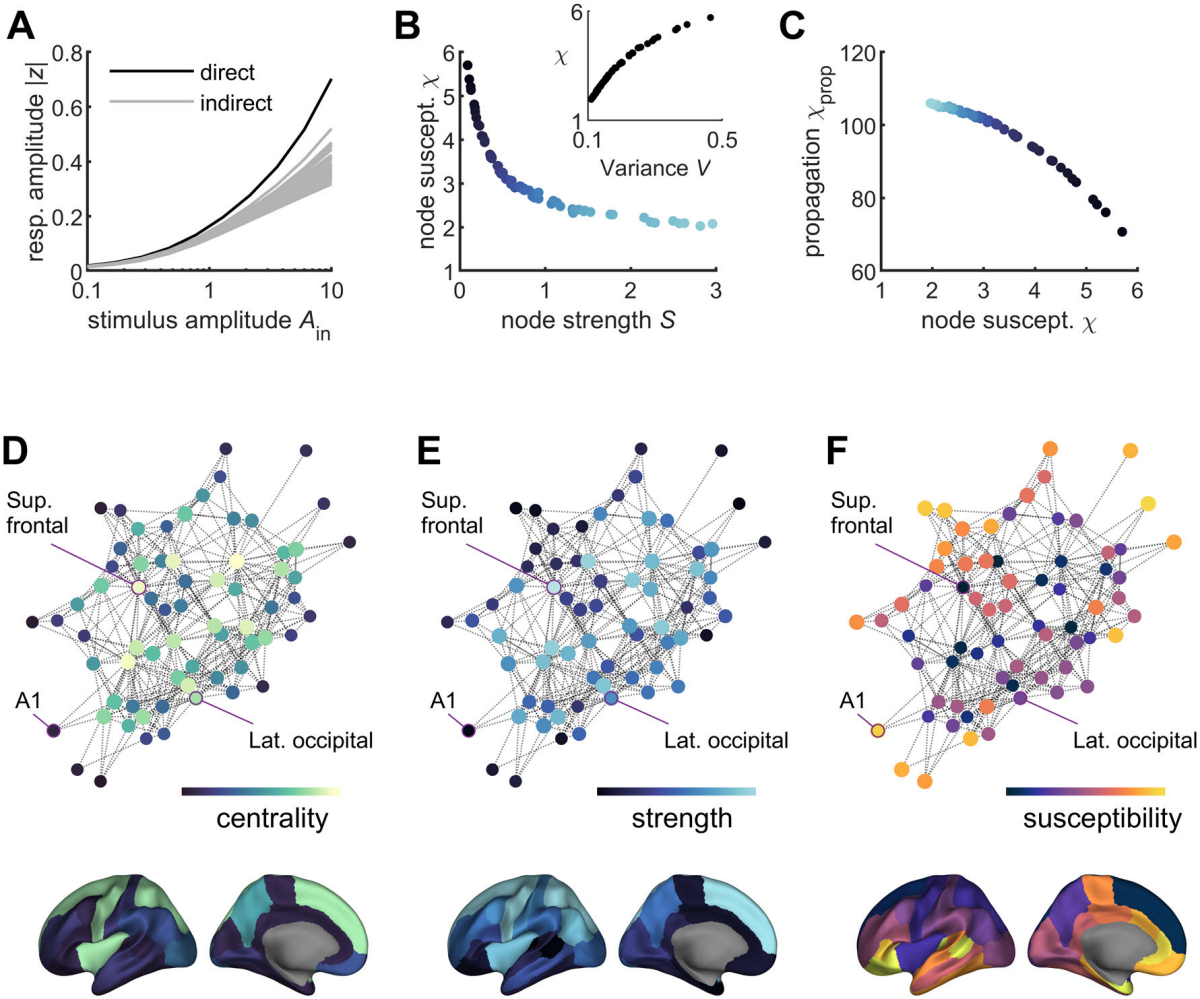
Susceptibility to external stimuli and core-periphery structure. ***A***, An oscillating stimulus of amplitude $A_{\mathrm{in}}$ was imposed to the j-th node of the homogeneous Hopf model ( a=−0.1, g=15×‖W‖). The response of the directly stimulated node (black trace) and the responses of the indirectly stimulated nodes (gray traces) were examined as a function of the stimulus amplitude. ***B***, The susceptibility $\chi_{j}$ of a stimulated node j decreased as a function of the node's connection strength. Inset: susceptibility versus variance. ***C***, The effect of stimulating node j on the other nodes of the network was given by the propagation susceptibility $\chi_{\mathrm{prop}}$, which is a decreasing function of the node's susceptibility. In other words, though strongly connected nodes transmitted more the stimulus to the other nodes, they responded less to incoming stimuli. ***D–F***, The graph representing the connectome was plotted using force-directed layout, i.e., using attractive forces between strongly connected nodes and repulsive forces between weakly connected nodes, allowing to visualize the center and the periphery of the graph. In panels ***D***, ***E***, and ***F***, the nodes were colored as a function of their betweenness centrality, connection strength, and susceptibility, respectively. Nodes that take a central role in the graph had high strength and low susceptibility, while nodes lying at the graph's periphery had a lower strength and a higher susceptibility. The nodes corresponding to the superior frontal region, the primary auditory cortex (A1, i.e., transverse temporal region), and the lateral occipital cortex are indicated in the graph. The brain surfaces show the spatial maps of betweenness centrality, connection strength, and susceptibility, in the DK parcellation. The model parameters used in this figure were those corresponding to the star in Figure 4*C*,*D*.

The regional variation of timescales has been associated to the functional specialization of brain regions, with fast fluctuations in sensory regions to rapidly encode stimuli and slower dynamics in higher-association regions allowing to integrate the oncoming information (Honey et al., 2012). Similarly, the functional implications of the hierarchy of variances observed here can be understood in terms of the response to an external stimulus and its propagation through the system. To study this, an oscillatory input of amplitude $A_{\mathrm{in}}$ was applied separately to each node of the Hopf model. The response of the nodes was quantified by the average modulus of their state variables during the stimulus, i.e., $\left\langle \left|z_{j}\right|\right\rangle$, for 1≤j≤N (see Materials and Methods). The response of the node directly receiving the stimulus increased as a function of the stimulus amplitude, as did, in a lesser extent, the response of the other nodes of the network (Fig. 8*A*). The total responses were quantified by summing over stimulus amplitudes the response of the stimulated node (node susceptibility, χ) and the average response of the other nodes (propagation susceptibility, $\chi_{\mathrm{prop}}$; see Materials and Methods). While the susceptibility of nodes was a decreasing function of the connection strength (Fig. 8*B*), their propagation capacity decreased with increasing susceptibility (Fig. 8*C*). This has consequences on the specialization of information processing of different parts of the network, namely, the center and the periphery of the network—which can be seen using graph visualization (Fruchterman and Reingold, 1991). Indeed, high-strength nodes that take a central role in the graph have a low susceptibility to incoming stimuli, while low-strength nodes lying at the graph's periphery have a higher susceptibility to external stimulus (Fig. 8*D–F*)—e.g., the primary auditory cortex lies at the periphery of the network and has low connectivity strength, but high susceptibility, while a more central region, such as the superior frontal region, exhibits the opposite characteristics. This implies that the propagation capacity of central nodes is counterbalanced by their low susceptibility.

## Discussion

The present study shows that the variance and temporal scales of fMRI resting-state fluctuations have opposite relationships with the structural connectivity: while more structurally connected brain regions presented activity fluctuations with longer timescales, their activity fluctuations presented lower variances. These coexistent and opposing structure–function relationships cannot be understood using simple linear dynamics, but they jointly emerge in two commonly used whole-brain models, the Hopf and Wilson–Cowan models, within specific parameter regions. This happens even when all nodes share the same intrinsic dynamics, demonstrating that hierarchical structure–function relationships can be explained by the interplay between connectivity and network state. These findings indicate that structure–function relationships are state dependent, therefore opening the possibility to be jointly used as biomarkers to characterize brain dynamics in different behavioral, vigilance, or conscious states, as well as in neuropsychiatric disorders—aligning with previous work reporting atypical neural timescales in autism (Watanabe et al., 2019) and modulation of neural timescales as a function of tasks, aging (Gao et al., 2020), attention (Zeraati et al., 2023), and behavioral demands (Manea et al., 2024). Additionally, the hierarchy of variances reflects different abilities to respond to and transmit external stimuli by different parts of the network, with larger responses at the network periphery than at the network core.

Previous reports using calcium imaging in zebrafish and fMRI in humans have shown that neurons/brain regions presenting high fluorescence/fMRI signal variance have low functional connections (Zarei et al., 2022). The negative correlation between variance and structural connection strength observed in the current f/dMRI data is consistent with these findings. Notably, as shown here, this structure–function relationship cannot be trivially explained by linear network propagation. In the Hopf model, this relationship is a consequence of diffusive coupling, while in the Wilson–Cowan model, it results from feedforward inhibition and varies based on the network's state, which can make the correlation between variance and connection strength either positive or negative. Additionally, state dependence is observed for the relationship between the timescale of activity fluctuations and the connection strength in both models. For the Wilson–Cowan model, the two opposite structure–function relationships jointly emerge in a balanced or inhibition-dominated parameter region that is close to the bifurcation where spirals lose stability and beyond which self-sustained oscillations appear—a scenario that is consistently observed in cortical activity (Okun and Lampl, 2008; Sanzeni et al., 2020). This is also consistent with previous studies suggesting that whole-brain dynamics are poised at the edge of instability (Ghosh et al., 2008; Aburn et al., 2012; Deco et al., 2013; Solovey et al., 2015). Recent research suggests that under the assumption of linear dynamics, sensory brain areas are closer to instability, while higher-order cortices move further away from it (Morales et al., 2023). Nevertheless, it is worth noting that this would imply a positive correlation between the hierarchy of variances and the hierarchy of timescales. In contrast, our data reveal that these hierarchies are inversely ordered, a pattern that only emerges in nonlinear models, thus highlighting the importance of nonlinearities.

It has been shown that the hierarchical organization of timescales not only correlates with the strength of structural connections but also to gradients in the spine density on pyramidal neurons (Elston, 2003, 2007; Chaudhuri et al., 2015), in gray matter myelination (Glasser and van Essen, 2011), and in the expression of synapses and cell-type receptor genes (Burt et al., 2018). Previous studies have modeled such gradients by incorporating them across the nodes of network, e.g., through differences in the strength of recurrent excitatory connections (Chaudhuri et al., 2015; Demirtaş et al., 2019; Mejías and Wang, 2022; Ding et al., 2024). In contrast, the present study shows that the observed opposing structure–function relationships can arise from the interplay between connectivity and network state, i.e., within specific parameter regions, even when intrinsic dynamics are either identical across all nodes or unstructured (i.e., random). Introducing a macroscopic gradient of myelination that modulates the intrinsic node dynamics, while suppressing connectivity-driven heterogeneity, results in structure–function relationships that cannot have opposing signs. This underscores that the observed structure–function relationships are driven by network effects. Future research might extend the models to include other gradients, such as the mRNA expression maps (Gao et al., 2020; Deco et al., 2021). Beyond this, an ambitious avenue for future research might be to investigate how gradients, connectivity, plasticity, and structure–function relationships coevolve and potentially interact during development to establish a functional hierarchy.

Like previous modeling studies (Chaudhuri et al., 2015; Demirtaş et al., 2019; Mejías and Wang, 2022; Ding et al., 2024), the present study uses connectome-based models to explore how structure–function relationships emerge in networks composed of a discrete set of nodes connected by structural links. Alternatively, recent findings have shown that a hierarchy of timescales can emerge in continuous models that apply a wave equation to the cortical surface (Pang et al., 2023). Future investigation could test whether the hierarchy of variances of the brain's spontaneous activity fluctuations also emerge in a model of propagating waves.

In addition, the present study concentrates on fluctuations around a stable equilibrium point, around which the noise can produce stochastic oscillations. In this regime, fluctuations can be treated linearly, allowing for the derivation of equations for network statistics (e.g., power spectral density, covariances, and autocorrelation functions). However, for certain model parameters, the dynamics may follow limit cycles, resulting in deterministic, self-sustained oscillations. In this latter case, linearization of fluctuations is not feasible and network statistics must therefore be studied through numerical stochastic simulations, limiting the systematic exploration of the parameter space. Building on previous work studying the hierarchy of timescale in Kuramoto networks of phase oscillators (Cocchi et al., 2016; Gollo et al., 2017), future research might explore the covariations of timescales and variances in the limit-cycle regime. Such investigation should pay special attention to both phase and amplitude interactions, phase response curves, and to potential stochastic resonances arising from the interplay between noise and quenched disorder due to heterogeneity. Indeed, moderate levels of noise have been shown to enhance synchronization between oscillators interacting through the connectome and exhibiting hierarchical properties (Pang et al., 2021).

Finally, previous studies on complex systems have established general principles of core-periphery network structures, showing that the periphery of the network tends to be more variable, evolvable, and plastic, while the network core supports the system's robustness and stability (Kitano, 2004; Csermely et al., 2013). This configuration with a stabilizing core and a flexible periphery was also reported in a seminal study on a large-scale model of neural mass oscillators interacting through the primate cortical connectome (Gollo et al., 2015). Core stability and peripheral variability have been observed experimentally in neural systems at different scales and with different techniques, including human fMRI activity during learning (Bassett et al., 2013), spontaneous calcium imaging in the mouse cortex (Betzel et al., 2019), and spiking activity in the auditory cortex of anesthetized rats (Ponce-Alvarez et al., 2020). Using methods from sloppy systems theory, it has been shown that while the cortical state is maintained by the activity of neurons that form a network core and relate to sensitive system parameters (“stiff dimensions”), stimulus-evoked responses are associated with the activity of neurons that form the network periphery and relate to insensitive system parameters (“sloppy dimensions”)—a configuration allowing the system to respond to stimuli without compromising the network's integrity (Ponce-Alvarez et al., 2020). In line with these findings, the present results indicate that the hierarchy of variances reflects a trade-off between stability and responsivity, with greater responsiveness at the network periphery, while the core ensures overall system stability. This, coupled with the hierarchy of timescales, results in a fast and responsive periphery alongside a slow and stable core, significantly influencing the brain's ability to integrate and segregate information. Indeed, such organization might be relevant for conscious experience, since a loss of core stability has been observed in low-levels states of consciousness (López-González et al., 2021).
